# Target-capture full-length double-stranded cDNA long-read sequencing through Nanopore revealed novel intron retention in patient with tuberous sclerosis complex

**DOI:** 10.3389/fgene.2023.1256064

**Published:** 2023-09-27

**Authors:** Hiroki Ura, Sumihito Togi, Yo Niida

**Affiliations:** ^1^ Center for Clinical Genomics, Kanazawa Medical University Hospital, Uchinada, Ishikawa, Japan; ^2^ Division of Genomic Medicine, Department of Advanced Medicine, Medical Research Institute, Kanazawa Medical University, Uchinada, Ishikawa, Japan

**Keywords:** RNA sequencing, next-generation sequencing, long-read sequencing, alternative splicing analysis, tuberous sclerosis complex

## Abstract

Tuberous sclerosis complex (TSC) is a relatively common autosomal dominant disorder characterized by multiple dysplastic organ lesions and neuropsychiatric symptoms caused by loss-of-function mutation of either *TSC1* or *TSC2*. The genetic diagnosis of inherited diseases, including TSC, in the clinical field is widespread using next-generation sequencing. The mutations in protein-coding exon tend to be verified because mutations directly cause abnormal protein. However, it is relatively difficult to verify mutations in the intron region because it is required to investigate whether the intron mutations affect the abnormal splicing of transcripts. In this study, we developed a target-capture full-length double-stranded cDNA sequencing method using Nanopore long-read sequencer (Nanopore long-read target sequencing). This method revealed the occurrence of intron mutation in the *TSC2* gene and found that the intron mutation produces novel intron retention splicing transcripts that generate truncated proteins. The protein-coding transcripts were decreased due to the expression of the novel intron retention transcripts, which caused TSC in patients with the intron mutation. Our results indicate that Nanopore long-read target sequencing is useful for the detection of mutations and confers information on the full-length alternative splicing of transcripts for genetic diagnosis.

## Introduction

The large majority of human genes are transcribed as pre-mRNAs that include exons and introns and then are spliced by the spliceosome to remove the introns and produce mature mRNA (Shi, 2017). The various mRNA products that encode structurally and functionally different protein isoforms are generated by post-transcriptional alternative splicing (Bush et al., 2017). Genome-wide studies and mass spectrometry analyses estimate that approximately 90% of human genes undergo alternative splicing and that approximately 40% of 20,000 human protein-coding genes generate multiple protein isoforms (Pan et al., 2008; Wang et al., 2008; Kim et al., 2014). Several studies suggested that alternative splicing occurs simultaneously in multiple genes during development and cellular differentiation (Martinez et al., 2012; Dillman et al., 2013; Singh et al., 2014). On the other hand, inappropriate splicing causes various human diseases, including several types of cancer (Venables, 2004; Srebrow and Kornblihtt, 2006).

RNA sequencing (RNA-seq) is a powerful tool for alternative splicing analysis and quantification of gene expression (Byron et al., 2016). Currently, short-read sequencing is the most popular sequencing technology. Short-read sequencing is a well-supported method for transcriptomics and is both high-throughput and affordable (Mortazavi et al., 2008). However, the short-read sequencing method struggles to determine how these features are combined into isoforms due to the fragmentation of RNA prior to sequencing. Long-read sequencing methods commercialized by PacBio (Pacific Biosciences) and Nanopore (Oxford Nanopore) have a distinct advantage over short-read sequencing methods because long-read sequencing can capture full-length transcripts (Rhoads and Au, 2015; Kono and Arakawa, 2019).

Tuberous sclerosis complex (TSC, OMIM #191100 and #613254) is an autosomal dominant inherited genetic disorder characterized by multiple organ lesions, facial angiofibroma, epilepsy, neuropsychiatric manifestations, and development of hamartomas throughout the body, particularly in the brain, skin, heart, and kidneys. The estimated morbidity of TSC in the population is about one in 6,000 to 10,000, of whom approximately two-thirds are sporadic, with the remainder segregated in families (Northrup et al., 2021). TSC is caused by loss-of-function mutations in *TSC1* or *TSC2* genes, which act as tumor growth suppressors and encode the proteins hamartin and tuberin and have been found to be responsible for the mTOR pathway (van Slegtenhorst et al., 1997; European Chromosome 16 Tuberous Sclerosis Consortium, 1993). The mutation patterns of *TSC1* and *TSC2* are diverse, with no mutational hotspots. Furthermore, the different types of mutations, including large deletions and deep intronic mutations, which generate abnormal splicing variants, were included in a non-negligible proportion. Owing to genetic heterogeneity, the clinical phenotype of the disease presents high variability, thus making the genetic diagnosis of TSC difficult. For that reason, it is required to analyze the full-length transcripts of *TSC1* and *TSC2* genes for the genetic diagnosis of TSC.

Here, we captured *TSC1* and *TSC2* full-length transcripts using a target-specific capture method and then performed long-read sequencing using Nanopore sequencer. We describe the target-capture full-length double-stranded cDNA sequencing using Nanopore long-read sequencer (Nanopore long-read target sequencing) method for accurate splicing variant detection and mutation detection.

## Materials and methods

### Patient and sample

A 38-year-old female was suspected of having tuberous sclerosis complex (TSC), and surveillance examination revealed that she met the clinical diagnostic criteria of definite TSC (Northrup et al., 2021). She had 17 hypomelanotic macules, three facial angiofibromas, five ungual fibromas, more than five cortical tubers, more than five subependymal nodules, and three renal angiomyolipomas. She also suffered from uncontrolled epilepsy and intellectual disability (FIQ is 62 by WAIS-III). After genetic counseling, peripheral blood was collected from the patient. Written informed consent was obtained, and the Ethics Review Board of Kanazawa Medical University approved the study design (G111).

### Genomic DNA and total RNA extraction

Genomic DNA and total RNA from peripheral blood mononuclear cells were obtained from a patient with TSC. Genomic DNA was extracted using a rapid extraction method (Lahiri and Schnabel, 1993), and total RNA was extracted using TRIzol reagent (Thermo Fisher Scientific) according to the manufacturer’s instructions, as described previously (Togi et al., 2021). The amount and optical density (A260/280 ratio) of genomic DNA and Total RNA were measured using Nanodrop (Thermo Fisher Scientific). The RNA integrity number (RIN) was measured using TapeStation 4200 with RNA ScreenTape (Agilent Technologies).

### Nanopore long-read target sequencing

According to the manufacturer’s instruction, the full-length double-strand cDNA was synthesized from the total RNA using the SMARTer-Seq HT Kit (Takara Bio USA) and then hybridized to *TSC*1 and *TSC2* gene-targeted capture probes, as described previously (Ura et al., 2021a). The hybridized double-strand cDNA was captured using streptavidin-coated beads. The captured cDNA was amplified over 14 cycles, and then, the ligation sequencing kit (SQK-LSK109) was used to create the RNA-seq library for Nanopore long-read sequencer (Oxford Nanopore Technologies). Library concentration was measured using Qubit. The libraries were loaded and sequenced on MinION flow cells. Base calling was performed concurrently with the MinION software (MinKNOW, v22.10.7). Only passed reads as designated by the software were used for subsequent analyses.

### Nanopore target splicing analysis

To compare the repertoires of *TSC1* and *TSC2* transcripts between the patient and two controls, the number of reads on target genes (*TSC1* and *TSC2*) in FASTQ files were made the same between the patient and two controls using the SeqKit software (version 0.13.2; Shen et al., 2016; Supplementary Table S1). Then, only the reads carrying poly(A) tail were extracted using SeqKit. The trimmed FASTQ files were aligned to the reference human genome (hg38) using Minimap2 (Version 2.18; Li, 2018). The FLAIR software (v1.5.0; Tang et al., 2020) was used to identify the registered and novel transcripts and to quantify the registered and novel transcripts with default settings. Next, we identified candidate coding regions of the detected transcripts using the TransDecoder program implemented in the Trinity software distribution (Grabherr et al., 2011). For analysis and interpretation, we used SAMtools (v1.9; Li et al., 2009), BEDTools (v2.27.1; Quinlan and Hall, 2010), Integrative Genomics Viewer (IGV 2.4.13; Thorvaldsdottir et al., 2013), and analysis approaches described previously (Ura et al., 2021b).

### Nanopore variant calling

As in Nanopore target splicing analysis, the FASTQ files were trimmed using SeqKit, and then, the trimmed FASTQ files were aligned with the reference human genome (hg38) using Minimap2 with default settings. The variants were identified using Medaka variant calling using the reference human genome (hg38) with default settings. For variant annotation, we used the following databases: SnpEff (version SnpEff 4.3t; Cingolani et al., 2012), dbSNP (version 151; Sherry et al., 2001), ClinVar (Landrum et al., 2018), Human Genetic Variation Database (HGVD; Higasa et al., 2016), and ToMMo (Version 3.5; Tadaka et al., 2018). The coverage of *TSC*1 and *TSC2* transcripts in genomic regions was calculated using RSeQC (version 3.0.1; Wang et al., 2012).

### Multiplex long-amplicon sequencing

Long-range PCR-based next-generation sequencing (NGS), also known as multiplex long-amplicon sequencing (MuLAS), was performed at the *TSC*2 genomic region, as previously described (Togi et al., 2021). In brief, a set of very-long-range PCR products (about 20 kb each) covering the *TSC2* entire gene locus was amplified by KOD One (TOYOBO) touchdown PCR. The PCR primer sequences used in this study are shown in Table 1. The NGS library was prepared from purified very-long-range PCR products using the Nextera Flex DNA Kit (Illumina) according to the manufacturer’s protocol.

**TABLE 1:** TSC long PCR primers.

Primer set	Primer name	Primer sequence	Primer position	Product size (bp)	Final primer concentration (μM)	Multiplex group
Multiplex long PCR primers			hg38			
T1-1	TSC1_1-2F	ACA​TCG​TCA​GTT​ATG​AGT​GGA​AGA​GCC​TC	chr9:132944777-132944749	9884	0.1375	A
	TSC1_1-2R	GAC​TAA​TCC​CTT​CAT​GCC​ATA​GAT​GGT​CC	chr9:132934894-132934922			
T1-2	TSC1_In2-F	GAG​GAA​GAT​GGG​AGG​GAA​TTA​TCT​TGC	chr9:132935006-132934980	6068	0.06	B
	TSC1_In2-R	GGG​CCA​CTA​CCA​AAC​TGA​GAA​AAA​GG	chr9:132928939-132928964			
T1-3	TSC1_3-8F	GGA​TAC​CTC​CCT​GTA​GCC​AGT​GGT​ATT​TG	chr9:132929527-132929499	8728	0.105	A
	TSC1_3-8R	TCA​GTC​TTA​CCC​TCA​GTT​CCA​CTC​TCC​AC	chr9:132920800-132920828			
T1-4	TSC1_In8-F	ATG​TGA​TCT​AGG​AGG​TAA​TCC​CTG​TTG​AGG	chr9:132921313-132921284	8941	0.2	B
	TSC1_In8-R	GCC​ATC​TTC​ATA​TGA​GGC​TTC​TGT​GG	chr9:132912373-132912398			
T1-5	TSC1_9-15F	CCT​TGA​TAG​GAG​ACC​TTA​AGT​CAG​CCT​CAG	chr9:132912861-132912832	8327	0.115	A
	TSC1_9-15R	TTA​GAT​CAC​TTC​CTT​GTG​GTC​AAA​ATC​TGC	chr9:132904535-132904564			
T1-6	TSC1_16-23F	CCT​CTA​ACT​CTC​TGT​GGA​CCT​GGA​GTT​TG	chr9:132904611-132904583	8671	0.12	B
	TSC1_16-23R	GGG​GGA​AAG​GAA​GAA​AGT​AAA​GCT​ACT​GAG	chr9:132895941-132895970			
T1-7	TSC1_3UTR-F	CCT​TTC​TGA​GAG​CCT​AAA​GAC​AGA​ACT​GG	chr9:132896394-132896366	7882	0.0625	A
	TSC1_3UTR-R	AGG​GCC​ACT​AAA​CAC​AAG​TGT​AGA​CAG​C	chr9:132888513-132888540			
T2-1	TSC2_2-7F	GCT​GTA​GTT​GAG​TTC​TCC​CAG​GGA​GTG	chr16:2048444-2048470	7941	0.1	B
	TSC2_2-7R	GAC​TCC​TGA​GGC​TCA​GAG​AGA​CCG​AG	chr16:2056384-2056359			
T2-2	TSC2_8-16F	TGA​GCC​TCA​GGA​GTC​CCC​CAT​GTA​AG	chr16:2056370-2056395	9751	0.075	A
	TSC2_8-16R	ACT​GGT​ATG​CTC​AGG​AGG​TTC​TCA​AGC	chr16:2066120-2066094			
T2-3	TSC2_In16-F	CAT​TTT​GGT​TTC​TGC​ACA​GTC​ACT​CG	chr16:2064766-2064791	6288	0.09	B
	TSC2_In16-R	GCC​AAC​ATC​TAT​AGC​GCA​AAC​TCA​GC	chr16:2071053-2071028			
T2-4	TSC2_17-26F	GAC​TCT​TCC​TCA​CCT​GTT​GAT​GAC​TGC	chr16:2070224-2070250	8948	0.055	A
	TSC2_17-26R	AGA​AGA​CGT​ATC​GAG​CCA​TCA​TGT​CC	chr16:2079171-2079146			
T2-5	TSC2_27-42F	ACG​CCC​TGT​TGG​GGT​CTT​TCC​GAG	chr16:2078905-2078928	9847	0.044	B
	TSC2_27-42R	CGC​ACC​AAG​CAG​ACA​AAG​TCA​ATA​AAA​GAG	chr16:2088751-2088722			
5'UTR long PCR primers						
T1-5UTR	TSC1_5UTR-F	TCA​AGA​TGA​AGC​CTT​TGA​CAA​CAT​TCC	chr9:132960451-132960477	20164	0.15	
	TSC1_5UTR-R	GGT​TTC​AAG​CTG​CGT​CCA​TAA​ATT​ACC	chr9:132940314-132940340			
T2-5UTR	TSC2_5UTR-F	GTT​TGT​CTT​TGG​TGA​AGG​ATG​GAG​AGG	chr16:2032541-2032567	20222	0.15	
	TSC2_5UTR-R	TAA​AGC​TGC​CAG​AAT​CAG​TCT​CAC​TCG	chr16:2052762-2052736			
Full-length long RT-PCR primers						
T1-RT	TSC1_RT-F	GAA​CCT​TCA​GAA​CCT​GTA​GCA​C	NM_000368.5:c.-59_-38	3589	0.2	
	TSC1_RT-R	CAA​TAT​GCA​AGT​TAA​CAC​TGA​TTG​ACC	NM_000368.5:c.*9_*35			
T2-RT	TSC2_RT-F	GGGAGGGGTTTTCTGGTG	NM_000548.5:c.-32_-15	5505	0.2	
	TSC2_RT-R	CTG​ACA​GGC​AAT​ACC​GTC​CAA​G	NM_000548.5:c.*28_*42			
Sanger sequencing and CHIPS primers						
TSC2	Tub-37S	CAG​CAC​TGG​CCC​CAC​AAA​CCC				
	Tub-37AS	TGC​CAC​CAA​CCC​GGA​CAC​AGC				

### CHIPS and Sanger sequencing

To verify the detected variant, CEL nuclease-mediated heteroduplex incision with polyacrylamide gel electrophoresis and silver staining (CHIPS) analysis and direct DNA sequencing were performed by Sanger sequencing, as described previously (Niida et al., 2015; Ura et al., 2020).

### Minigene assay

For gene cloning, a region including exon 37, intron 37, and exon 38 of the *TSC*2 gene was PCR amplified from the patient’s genomic DNA using a set of primers: forward primer 5′-TCA​AGC​GAA​TTC​ATG​AGC​AAC​AGC​GAG​CTC​GCC​ATC​CTG​TCC-3′ and reverse primer 5′-ATG​ACC​GGT​GGA​TCC​GCC​TTG​ATG​GTG​CCA​AGC​TTG​AAG​TCC​TC-3′ and KOD-Plus-Neo DNA polymerase (TOYOBO; Figure 6A). The PCR product was cloned into pAcGFP1-N1, which was digested by BamHI and EcoRI restriction enzymes using the In-Fusion HD Cloning Kit (TaKaRa) according to the manufacturer’s protocol. The presence or absence of mutation [NM_000548.3:c.4850-2A>G p.(Ala1617GlyfsTer24)] was confirmed by Sanger sequencing.

The 1383D6 human-induced pluripotent stem cell (hiPSC) line was cultured in StemFit medium on iMatrix 511 (TaKaRa)-coated plates at 37 C in 5% CO_2_. The hiPSCs were passaged as clump with TrypLE Select (Life Technologies) at a ratio of 1:6 every 4–5 days. Cultured hiPSCs were seeded in a 24-well plate, and Minigene plasmids were added using the Lipofectamine Stem Transfection System (Thermo Fisher Scientific) according to the manufacturer’s protocol. After 3 days, the transfected hiPSCs were harvested, and the total RNA of the transfected hiPSCs was extracted. cDNAs from WT plasmid-transfected hiPSCs and mutant plasmid-transfected hiPSCs were synthesized using the PrimeScript RT-PCR Kit (TaKaRa) according to the manufacturer’s protocol. The PCR was performed using a set of primers: forward primer 5′-TCA​AGC​GAA​TTC​ATG​AGC​AAC​AGC​GAG​CTC​GCC​ATC​CTG​TCC-3′ and reverse primer 5′-CAT​TCA​GCT​CGA​TCA​GGA​TGG​GCA​C -3′and KOD-Plus-Neo DNA polymerase. The concentration of bands was measured by TapeStation 4200 with D1000 ScreenTape.

## Results

### Nanopore long-read target sequencing

**FIGURE 1:**
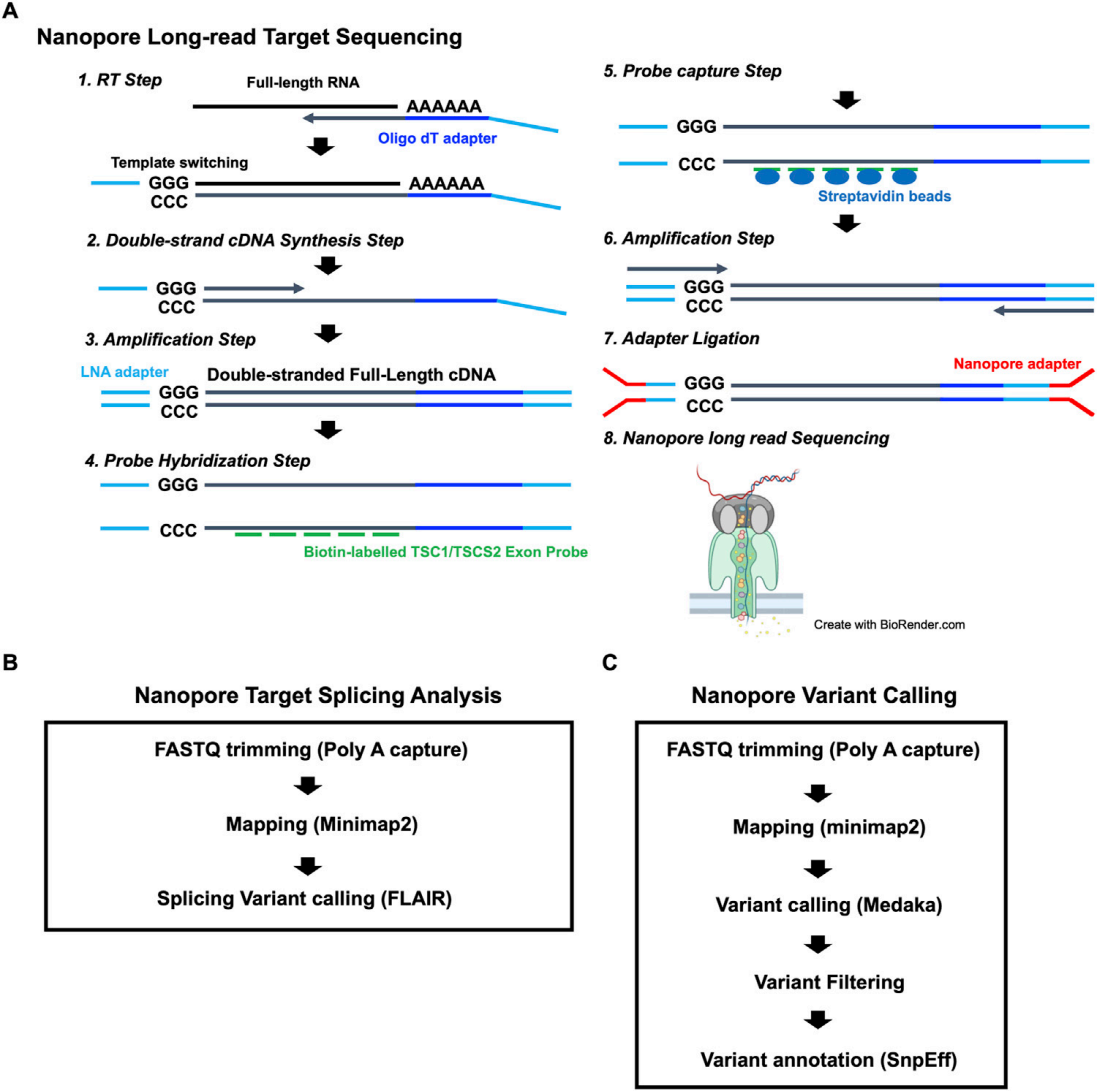
**(A)** Workflow for the Nanopore long-read target sequencing method. The figure was created using biorender.com. **(B)** Workflow for Nanopore target splicing Analysis. **(C)** Workflow for Nanopore variant calling.

**FIGURE 2:**
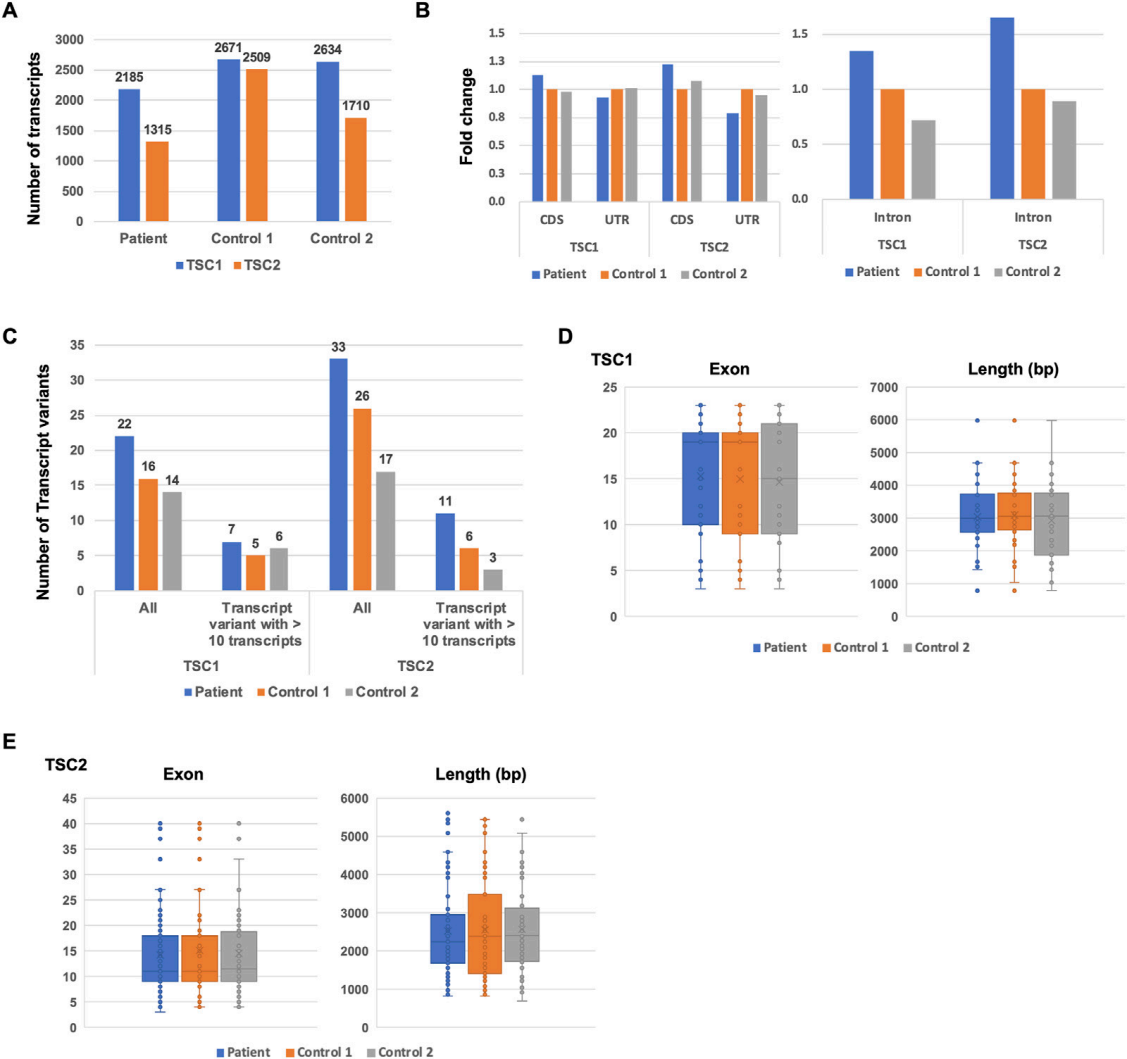
Comparison of *TSC1* and *TSC2* transcript repertoires between the patient and controls. **(A)** Number of *TSC1* and *TSC2* transcripts. **(B)** The fold-change of coverage in *TSC1* and *TSC2* genomic regions. The coverage data were normalized to control 1. **(C)** Number of transcript variants of *TSC1* and *TSC2* transcripts. The number of *TSC1* and *TSC2* transcript variants with 10 or more of the same type of transcript variant. **(D)** Number of exons in *TSC1* transcripts. Length of *TSC1* transcripts. The *t*-test showed no statistical difference (*p*-value > 0.5) between the patient and controls. **(E)** Number of exons in *TSC2* transcripts. Length of *TSC2* transcripts is shown. The *t*-test showed no statistical difference between the patient and controls (*p*-value > 0.5).

To identify the putative pathogenic mutations in tuberous sclerosis complex (TSC) patients, we developed a target-capture full-length double-stranded cDNA sequencing method using Nanopore long-read sequencer (Nanopore long-read target sequencing; Figure 1). To enrich the TSC-causing genes (*TSC1* and *TSC2*), the full-length double-stranded cDNA generated by the SMARTer method was captured using biotin-labeled TSC1/TSC2 exon probes (Figure 1A). Then, the captured full-length cDNA was amplified and adapter-ligated for Nanopore long-read sequencing. After sequencing, we performed the splicing analysis and variant calling (Figures 1B, C). To accurately analyze the transcript, the same number of reads that have poly(A) tail were extracted in the TSC patient and controls (Figure 1B). The transcript number, coverage, exon number, and transcript length were compared between the TSC patient and controls (Figure 2). The number of *TSC1* and *TSC2* transcripts in the patient was slightly fewer than that in controls (Figure 2A). The coverage of CDS and UTR in *TSC1* was almost the same between the TSC patient and controls (Figure 2B). The ratio of *TSC2* UTR coverage in the patient was slightly lower than that in controls. However, the coverage of intron in *TSC1* and *TSC2* was about 1.5-fold higher in the TSC patient. The number of *TSC1* transcript variants was almost the same between the TSC patient and controls (Figure 2C). The number of *TSC2* transcript variants in the patient was higher than that in controls. The difference between the TSC patient and controls in the transcript variants expressing more than 10 transcripts was smaller than in the total transcript variants, indicating that the number of low-expression transcript variants was increased in the TSC patient. These results suggest that the *TSC2* alternative splicing variants, including intron, might be produced in the TSC patient. The number of exons and the length of *TSC1* and *TSC2* transcripts were similar between the TSC patient and controls (Figures 2D, E), indicating that the *TSC2* intron retention variants may not be a large proportion in *TSC2* transcripts.

### Repertoire of *TSC1* and *TSC2* transcripts

**FIGURE 3:**
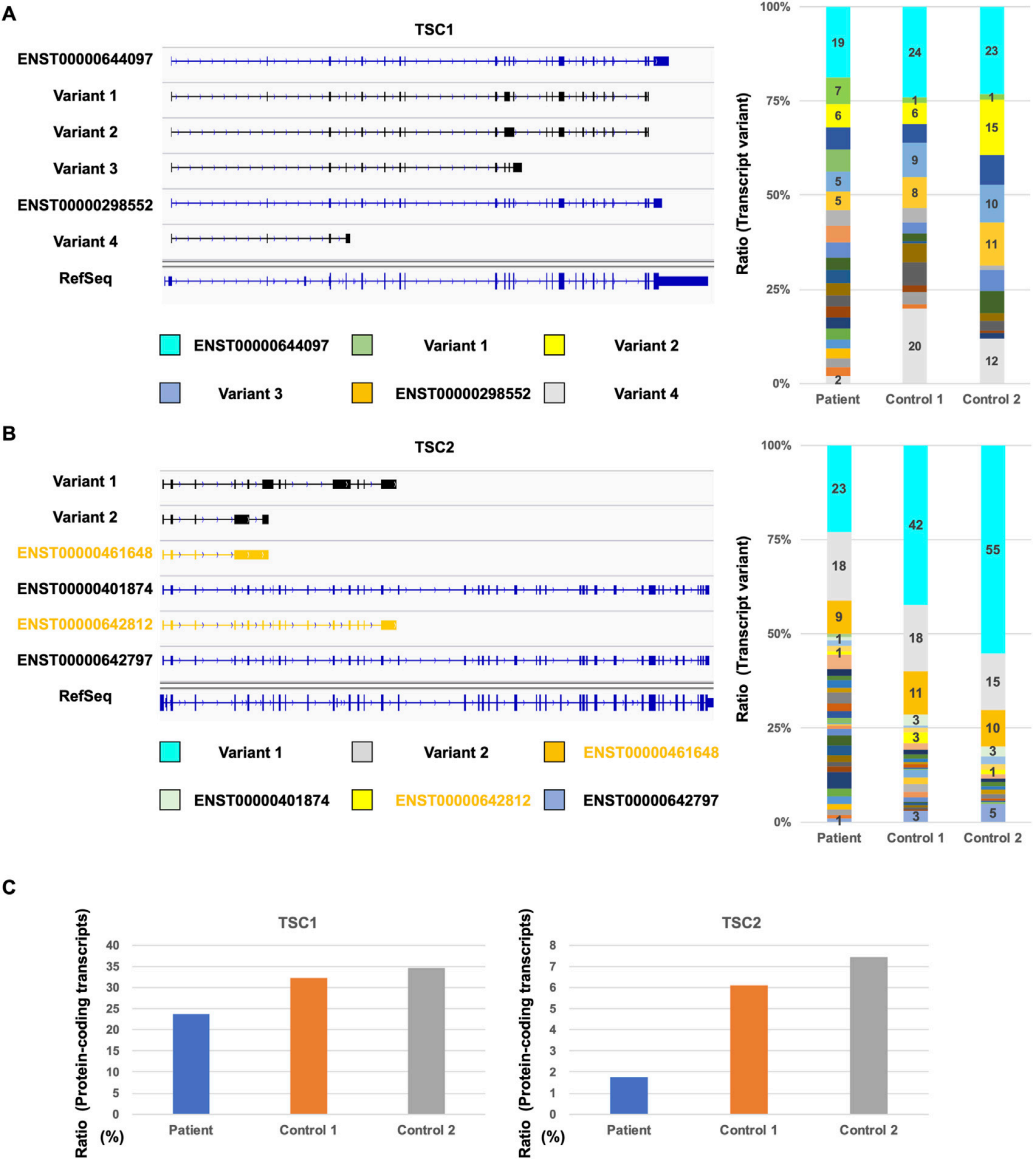
Comparison of *TSC1* and *TSC2* transcript repertoires between the patient and controls. **(A)** Repertoire of representative *TSC1* transcripts with a relatively high expression (left). Ratio of each *TSC1* transcript variants in all *TSC1* transcripts (right). **(B)** Repertoire of representative *TSC2* transcripts with a relatively high expression (left) and the ratio of *TSC2* transcript variants (right). Ratio of each *TSC2* transcript variant in all *TSC2* transcripts is shown. **(C)** Ratio of TSC1 and TSC2 protein-coding transcripts between the patient and controls.

Next, we investigated the repertoire of *TSC1* and *TSC2* transcripts to determine the number of TSC1 and TSC2 protein-coding transcripts. Although the database-registered transcripts, such as ENST00000644097 and ENST00000298552, were expressed in the patient and controls, many non-registered transcript variants were also expressed (Figure 3A). Each non-protein-coding transcript, such as variant 1, variant 2, variant 3, and variant 4, was expressed at different rates in the patient and controls. The ratio of ENST00000644097 and ENST00000298552, which are the protein-coding transcripts, in the patient was lower than that in controls (Figure 3C). Similar to *TSC1*, the registered transcripts, such as ENST00000461648, ENST00000401874, ENST00000642812, and ENST00000642797, were expressed, and many non-registered transcript variants were expressed in the patient and controls (Figure 3B). Surprisingly, the majority of the expressed transcripts were occupied by non-registered transcripts. Moreover, the ratio of the protein-coding transcripts, such as ENST00000401874 and ENST00000642797, was less than 10% in controls (Figure 3C). The ratio of the protein-coding transcripts in the TSC patient was less than one-third of that in controls. These results suggest that the TSC2 protein-coding transcripts were decreased in the patient.

### Nanopore variant calling

**FIGURE 4:**
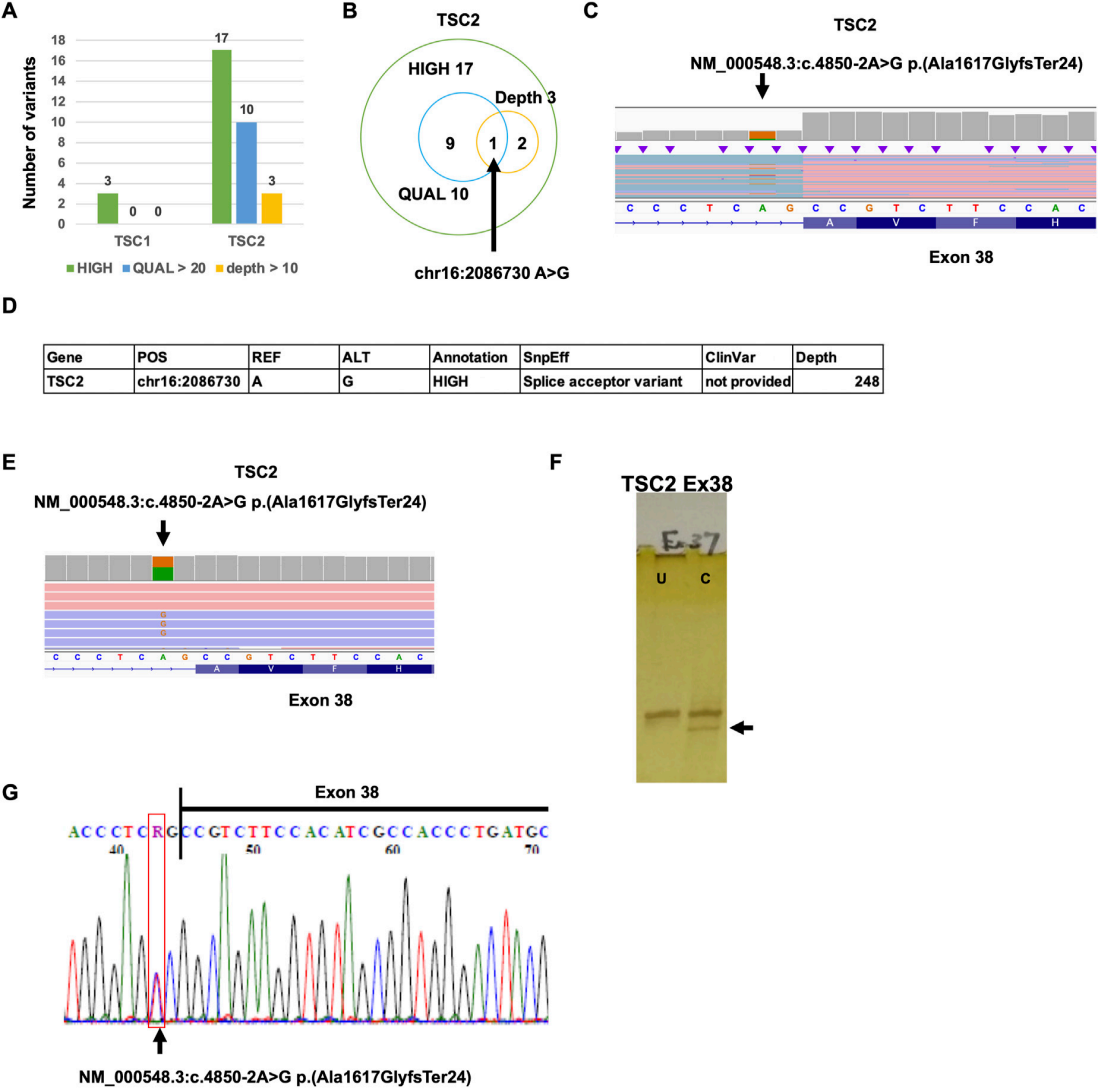
Detection of *TSC1* and *TSC2* variants. **(A)** Number of *TSC1* and *TSC2* variants detected by Nanopore variant calling. **(B)** Venn graph of *TSC2* variants. **(C)** The mutation [NM_000548.3:c.4850-2A>G p.(Ala1617GlyfsTer24)] was detected by Nanopore variant calling on IGV. **(D)** Summary of validation results. **(E)** The mutation [NM_000548.3:c.4850-2A>G p.(Ala1617GlyfsTer24)] was detected by MuLAS on IGV. **(F)** CHIPS technology assay. The arrow head shows cleaved heteroduplex. U, uncut; C, cut by CEL nuclease. **(G)** Electropherograms of Sanger sequencing of the detected variant.

To identify the putative pathogenic mutations that cause a decrease in TSC2 protein-coding transcripts, we performed Nanopore variant calling (Figure 1C). To accurately detect the mutations, the reads that have poly(A) tail were extracted in the TSC patient, and variant calling was subsequently performed. The mutations classified as HIGH by SnpEff were three mutations in *TSC1* and 17 mutations in *TSC2* (Figure 4A). The mutations with more than 20 QUAL score values were 10 mutations in *TSC2*. The mutations that were detected by more than 10 depth values were three mutations in *TSC2*. There was only one mutation common to all three conditions (HIGH, QUAL > 20, and depth > 10; Figure 4B; Supplementary Table S2). This mutation is the heterozygous mutation in *TSC2* [NM_000548.3:c.4850-2A>G p.(Ala1617GlyfsTer24)] that causes aberrant splicing events in SnpEff prediction because the mutation is located at the splicing acceptor site (Figures 4C, D). To validate the detection accuracy of Nanopore variant calling, we performed the very-long-amplicon sequencing (vLAS) analysis (Figures 4D, E). The mutation classified as HIGH by SnpEff was only one mutation in the *TSC2* genomic region. The mutation was the same one detected in Nanopore variant calling (Figure 4D). Moreover, CEL nuclease-mediated heteroduplex incision with polyacrylamide gel electrophoresis and silver staining (CHIPS) technology and Sanger sequencing also confirmed the same mutation (Figures 4F,G). These results suggest that Nanopore variant calling accurately detected the putative mutation responsible for the aberrant splicing events.

### Detection of novel intron retention splicing variants

**FIGURE 5:**
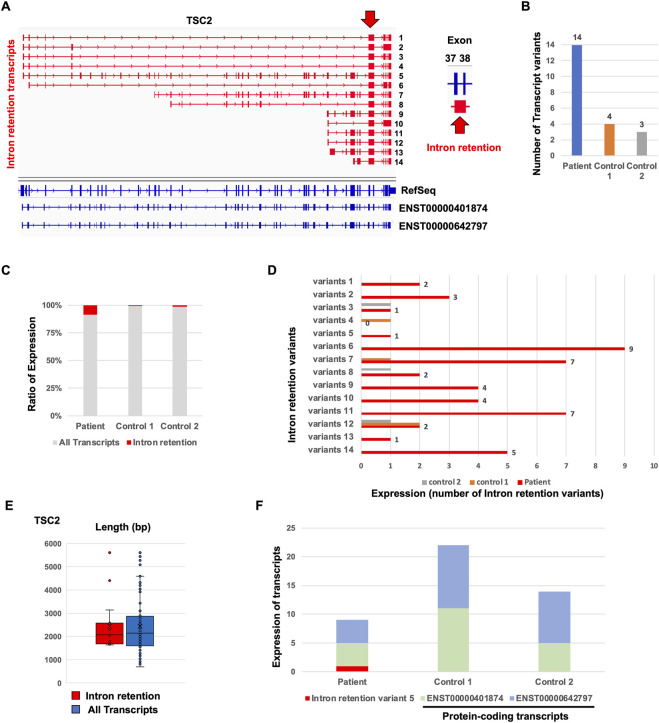
Comparison of *de novo TSC2* intron retention transcript variants between the patient and controls. **(A)** Repertoire of *de novo TSC2* intron retention transcript variants. **(B)** The number of *de novo TSC2* intron retention transcript variants. **(C)** Ratio of *de novo TSC2* intron retention transcript variants. **(D)** The number of each *de novo TSC2* intron retention transcript variants in the patient. **(E)** The length of *de novo TSC2* intron retention transcript variants. The *t*-test showed no statistical difference between the patient and controls (*p*-value > 0.9). **(F)** Expression of intron retention variant 5 and protein-coding transcripts (ENST00000401874 and ENST000000642797) between the patient and controls.

To identify the aberrant splicing events caused by the detected mutation, we performed splicing variant calling in Nanopore target splicing analysis (Figure 1B). The transcripts with the detected mutation caused the novel intron retention splicing events in the TSC patient (Figure 5A). The novel intron retention that occurred in intron 37 has the putative pathogenic mutation detected by Nanopore variant calling. TransDecoder (finding coding regions within transcripts) analysis indicated that the transcripts with the intron retention fail to produce the normal TSC2 protein due to the frameshift. The repertoire of *TSC2* transcript variants in the TSC patient was relatively higher than that in controls (Figure 5B). Furthermore, the ratio of the transcripts with intron retention in the patient was relatively higher than that in controls (Figure 5C). These results indicated that the *TSC2* transcript repertoire in the TSC patient with the putative pathogenic mutation contains relatively higher transcripts with novel intron retention than controls. The particular intron retention variant was expressed, and most of the variants were expressed evenly (Figure 5D). The length of intron retention variants was almost the same as that of all *TSC2* transcripts (Figure 5E). The expression of protein-coding transcripts (ENST00000401874 and ENST000000642797) in the TSC patient was lower than that in controls (Figure 5F). ENST00000401874 and ENST000000642797 have the same coding region but not the transcription start site (TSS). Although the intron retention variant 5 has the same coding region of ENST00000401874 and ENST000000642797, the transcript caused the intron retention in intron 37. The expression of ENST00000401874 and ENST000000642797 in the patient was clearly decreased compared to the controls due to the expression of the novel intron retention variant 5 (Figure 5F). These results suggested that the novel intron retention was detected in the TSC patient.

### Confirmation of novel intron retention caused by intron mutation using Minigene assay

**FIGURE 6:**
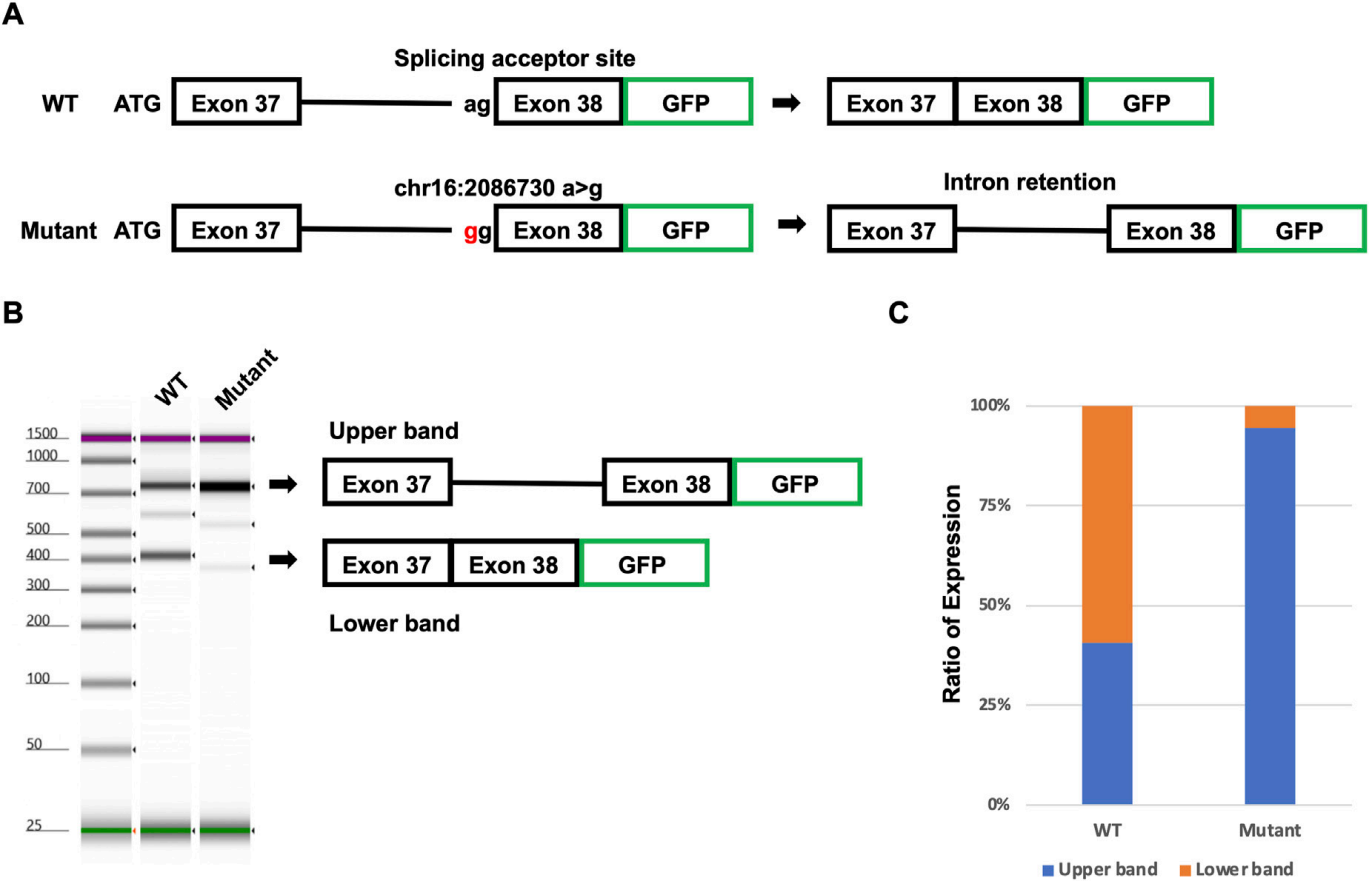
Confirmation of putative mutation using the Minigene assay. **(A)** Model of the Minigene assay. Mutant plasmid has mutation [NM_000548.3:c.4850-2A>G p.(Ala1617GlyfsTer24)]. **(B)** Result of TapeStation. The upper arrow head indicates intron retention transcripts. The lower arrow head indicates spliced transcripts. **(C)** Ratio of intron retention transcripts and spliced transcripts.

To confirm the novel intron retention caused by the detected putative pathogenic intron mutation, we performed the Minigene assay. Wild-type (WT) and mutant minigenes covering from exon 37 to exon 38, including intron 37, were generated (Figure 6A). The minigenes were transfected into human-induced pluripotent stem cells (hiPSCs), and the splicing was analyzed (Figures 6B, C). The WT minigene transfected into hiPSCs resulted in two distinct products of 728 and 376 bp (Figure 6B). The upper band (728 bp) represents the intron retention variant, and the lower band (376 bp) represents the correctly spliced transcript. Unlike the WT minigene, the mutant minigene transfected into hiPSCs resulted in almost one upper band. In the WT minigene, around 60% of transcripts were correctly spliced (Figure 6C). On the other hand, almost all transcripts caused intron retention in the mutant minigene carrying the detected putative mutation. These results suggested that the detected putative mutation [NM_000548.3:c.4850-2A>G p.(Ala1617GlyfsTer24)] will cause intron retention in the patient.

## Discussion

NGS-based transcriptome analysis can serve as a powerful tool for the quantification and detection of alternative splicing transcripts in human disease and development research. However, most studies showed only individual splicing events but not full-length transcripts due to short-read sequencing. It is possible to predict protein repertoire by investigation of entire transcript isoforms. Moreover, it is possible to quantify accurate expression at the transcript level. Here, we developed the target-capture full-length double-stranded cDNA sequencing using Nanopore long-read sequencer (Nanopore long-read target sequencing). This method revealed that the intron mutation in the *TSC2* gene produces the novel intron retention splicing transcripts which generate the truncated proteins. Nanopore long-read target sequencing will provide helpful information about the entire transcript in fundamental research and clinical diagnosis.

Nanopore long-read target sequencing is a combination of Nanopore target splicing analysis and Nanopore variant calling. In Nanopore target splicing analysis, various alternative splicing transcripts in both *TSC1* and *TSC2* were detected. Furthermore, intron retention variants which were caused by the detected putative pathogenic mutation in Nanopore variant calling were also detected. These results indicated that the Nanopore long-read target sequencing method is useful for clinical diagnosis. Surprisingly, almost all transcripts in both *TSC1* and *TSC2* were not registered in major databases, such as RefSeq and Ensembl, and were not protein-coding transcripts. Both *TSC1* and *TSC2* are not highly expressed in peripheral blood and are not functional except in starvation. It is possible to increase TSC1 and TSC2 protein-coding transcripts in starvation and in the TSC1 and TSC2 functionally working cells, such as nervous system cells (Hisatsune et al., 2021). Because it is difficult to generate the corrected peripheral blood from patients in starvation conditions, it is required to confirm the transcript repertoire of *TSC1* and *TSC2* in nervous system cells derived from patient-derived human-induced pluripotent stem cells (hiPSCs; Ura et al., 2023). Many studies suggested that the repertoire of transcripts depends on cell types (He et al., 2020; Heumos et al., 2023; Salataj et al., 2023). Using this method may help fundamental research because it is possible to capture unknown repertoires of transcripts in various cell types and in various developmental cells. Although Nanopore variant calling detected putative pathogenic mutation, the method detected around 20 mutations that may be false positive due to the quality of long-read sequencing. So far, the accuracy of long-read sequencers is lower than that of short-read sequencers, such as the Illumina sequencer (Koren et al., 2017). However, the accuracy of long-read sequencer is improving year by year due to the development of tools (Amarasinghe et al., 2020; Wang et al., 2021). In Oxford Nanopore Technology, the accuracy of sequencing reads is improving due to updated versions of the system to date and newer sequencing methods, such as 2D and 1D^2^ sequencing. For this reason, it is possible to improve the accuracy of the mutation detection in the Nanopore long-read target sequencing method using the newest version of the system and newer sequencing methods. Recently, Pacific Biosciences released a new highly accurate long-read sequencer called the PacBio Revio (Manuel et al., 2023). Because the Nanopore long-read target sequencing method can also be handled by changing PacBio Revio from Nanopore, the accuracy in the Nanopore long-read target sequencing method may be further improved using PacBio Revio. As described in this study, it is sufficiently detectable by setting the criteria as more than 20 QUAL and more than 10 depth values, as described in this study. Moreover, it is possible to detect more accurately by multiple Nanopore long-read target sequencing analyses, as described previously (Ura et al., 2020).

We used *TSC1* and *TSC2* capture probes to test the Nanopore long-read target sequencing method, although this method may be used for any commercial or laboratory-developed gene panels. So far, single-cell analyses, such as 10x Genomics chromium, analyze only quantification of transcripts due to sequencing only 3’ of mRNA (Danielski, 2023). The Nanopore long-read target sequencing method applies to single-cell transcriptome, including quantification of mRNA and alternative splicing analysis, using 10x Genomics chromium. The Nanopore long-read target sequencing method will provide the quantification of mRNA and alternative splicing information at the single-cell level. The Nanopore long-read target sequencing method is useful for the detection of mutations and confers information on full-length alternative splicing transcripts for the genetic diagnosis.

## Data Availability

The datasets presented in this study can be found in the Gene Expression Omnibus (GEO; https://www.ncbi.nlm.nih.gov/geo/). The accession number is GSE241410.
